# Basaloid squamous cell carcinoma of the maxillary sinus: Report of two cases in association with cathepsin K expression

**DOI:** 10.3892/ol.2013.1311

**Published:** 2013-04-17

**Authors:** MITSUAKI ISHIDA, HIDETOSHI OKABE

**Affiliations:** Department of Clinical Laboratory Medicine and Division of Diagnostic Pathology, Shiga University of Medical Science, Otsu, Shiga, Japan

**Keywords:** basaloid squamous cell carcinoma, maxillary sinus, cathepsin K

## Abstract

Basaloid squamous cell carcinoma (BSCC) is a rare variant of squamous cell carcinoma. The occurrence of BSCC in the nasal cavity is extremely rare. In the present study, two cases of BSCC occurring in the maxillary sinus are reported and the clinicopathological features and immunohistochemical characteristics of this rare tumor are discussed. Two patients, aged 85 (case 1) and 60 years (case 2), presented with nasal tumors and persistent nasal obstruction. In each case, the biopsy or resected specimen of the maxillary sinus tumor revealed an infiltrative proliferation of solid epithelial nests composed of basaloid cells exhibiting hyperchromatic nuclei without conspicuous nucleoli and scant cytoplasm. Mitotic figures were frequently observed and spherical hyalinized materials were present within the tumor nests. Immunohistochemically, the tumor cells exhibited diffuse positive immunoreactivity for p63 and perinuclear dot-like positivity for vimentin, leading to a final diagnosis of BSCC of the maxillary sinus. Furthermore, it was demonstrated for the first time in the two cases that cathepsin K, a cysteine protease with marked collagenolytic and elastolytic activities, was expressed in a diffuse manner. One patient (case 2) succumbed to multiple metastases, while the other (case 1) remains alive with the disease. In conclusion, it was demonstrated that cathepsin K was immunopositive in two cases of BSCC of the maxillary sinus and that it may be involved in tumor invasion by this highly aggressive carcinoma.

## Introduction

Basaloid squamous cell carcinoma (BSCC) is a rare variant of squamous cell carcinoma, which is characterized clinically by highly aggressive behavior and is histopathologically composed of basaloid and squamous components (1). BSCC was first reported by Wain *et al* in 1986 as a highly aggressive histopathological variant of squamous cell carcinoma occurring in the tongue, pharynx and larynx (2). Since this initial report, BSCC has been reported in various organs, including the esophagus, with the most common sites in the head and neck region being the oral cavity and larynx (1,3). The occurrence of BSCC in the nasal cavity is extremely rare and only 26 cases have been reported (3–9). In the present study, two additional cases of BSCC occurring in the maxillary sinus are reported and the clinicopathological features and immunohistochemical characteristics of this rare tumor are discussed. This study was approved by the ethics committee of Shiga University of Medical Science. Written informed consent was obtained from the patients.

## Case reports

### 

#### Case 1

**Figure 1:**
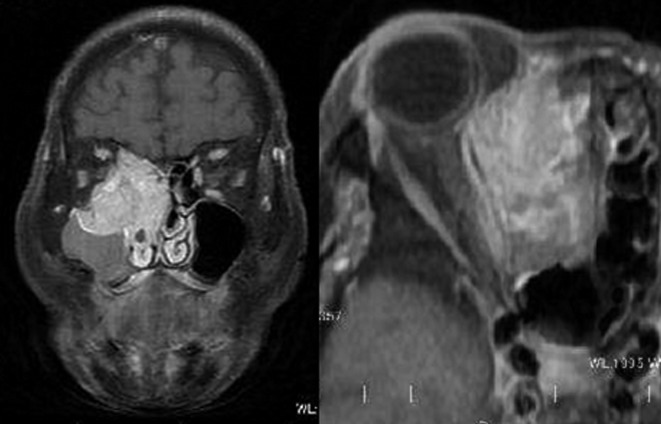
MRI showing a right-sided maxillary sinus tumor invading into the dura mater and orbit. MRI, magnetic resonance imaging.

An 85-year-old Japanese female patient presented with a nasal tumor and right-sided exophthalmos. Computed tomography (CT) and magnetic resonance imaging (MRI) scans revealed a right maxillary sinus tumor invading into the right ethmoid sinus, orbit and dura mater (Fig. 1). A biopsy specimen from the right maxillary sinus tumor indicated a diagnosis of BSCC (T4, N0, M0), and radiation therapy (58 Gy) with TS-1 administration was subsequently administered. CT and MRI scans following therapy showed that the tumor had contracted and a biopsy from the tumor showed a few residual degenerative carcinoma cells. The patient has survived with the disease for 10 months since the initial diagnosis.

#### Case 2

A 60-year-old Japanese male patient presented with an approximate three-month history of persistent nasal obstruction. CT revealed a right-sided maxillary sinus tumor with destruction of the surrounding bone tissues and dural invasion. A biopsy specimen from the maxillary sinus tumor revealed a poorly-differentiated carcinoma. Surgical resection of the maxillary sinus tumor was subsequently performed. Following surgery, the patient received chemotherapy. The post-operative course was uneventful, although local recurrence occurred one and a half years subsequent to the surgery. This was followed by the development of multiple liver and lung metastases to which the patient succumbed.

#### Materials and methods

The formalin-fixed, paraffin-embedded tissue blocks of the maxillary sinus specimens were cut into 3-*μ*m thick sections, then deparaffinized and rehydrated. Each section was stained with hematoxylin and eosin, then used for immunostaining. Immunohistochemical analyses were performed using an autostainer (Benchmark XT system, Ventana Medical Systems, Tucson, AZ, USA) according to the manufacturer’s instructions. The following primary antibodies were used: mouse monoclonal antibody against α-smooth muscle actin (alphasm-1; Novocastra Laboratories, Ltd., Newcastle upon Tyne, UK), mouse monoclonal antibody against cathepsin K (3F9; Abcam, Cambridge, UK), mouse monoclonal antibody against cytokeratin (AE1/AE3; DAKO Cytomation, Glostrup, Denmark), mouse monoclonal antibody against high molecular weight cytokeratin (34betaE12; DAKO Cytomation), mouse monoclonal antibody against epithelial membrane antigen (GP1.4; Novocastra), mouse monoclonal antibody against p63 (7JUL; Novocastra) and mouse monoclonal antibody against vimentin (VIM3B4; Novocastra).

### Histopathological results

#### Case 1

**Figure 2:**
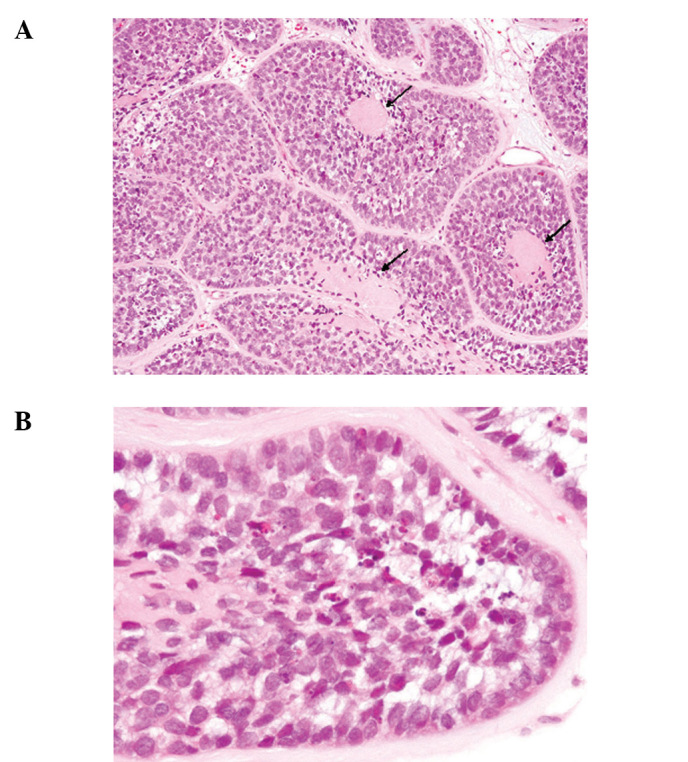
Histopathological findings of case 1. (A) Infiltrative proliferation of solid epithelial nests composed of basaloid cells. Spherical hyalinized materials were present within the tumor nests (arrows; hematoxylin and eosin staining; magnification, ×100). (B) The basaloid cells exhibit hyperchromatic nuclei without conspicuous nucleoli and scant cytoplasm. Numerous apoptotic bodies were observed (hematoxylin and eosin staining; magnification, ×400).

The biopsy specimen from the maxillary sinus tumor exhibited an infiltrative proliferation of solid epithelial nests composed of basaloid cells and the surface epithelium was eroded (Fig. 2A). These basaloid cells had a high nuclear/cytoplasmic ratio, hyperchromatic nuclei without conspicuous nucleoli and scant cytoplasm (Figs. 2A and B). Mitotic figures were scattered and apoptotic bodies were frequently observed (Fig. 2B). The characteristic histopathological finding was the presence of spherical hyalinized materials within the tumor nests (Fig. 2A, arrows). No keratinization was observed in the tumor cells.

#### Case 2

**Figure 3:**
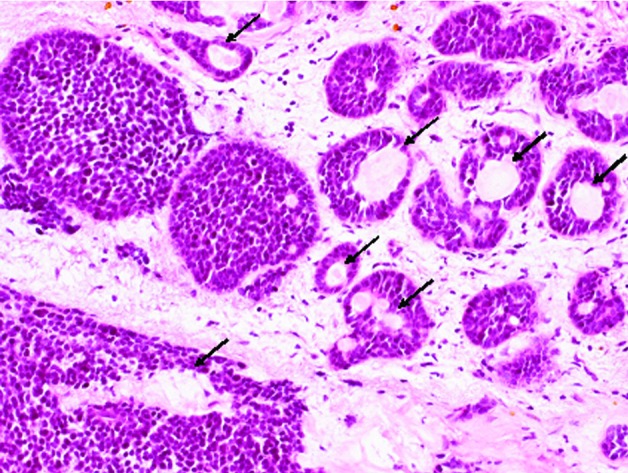
Histopathological findings of case 2. Infiltrative growth of basaloid cells exhibiting hyperchromatic nuclei without conspicuous nucleoli and scant cytoplasm. Spherical hyalinized materials were present within the tumor nests (arrows; hematoxylin and eosin staining; magnification, ×100).

The surgically resected specimen of the maxillary sinus showed an infiltrative proliferation of solid epithelial nests composed of the basaloid cells that had scant cytoplasm and hyperchromatic nuclei without conspicuous nucleoli (Fig. 3). Spherical hyalinized materials were present within the tumor nests (Fig. 3, arrows) and mitotic figures were scattered. Focal squamous differentiation, including individual keratinization and intercellular bridging, was also observed.

#### Immunohistochemical results

**Figure 4:**
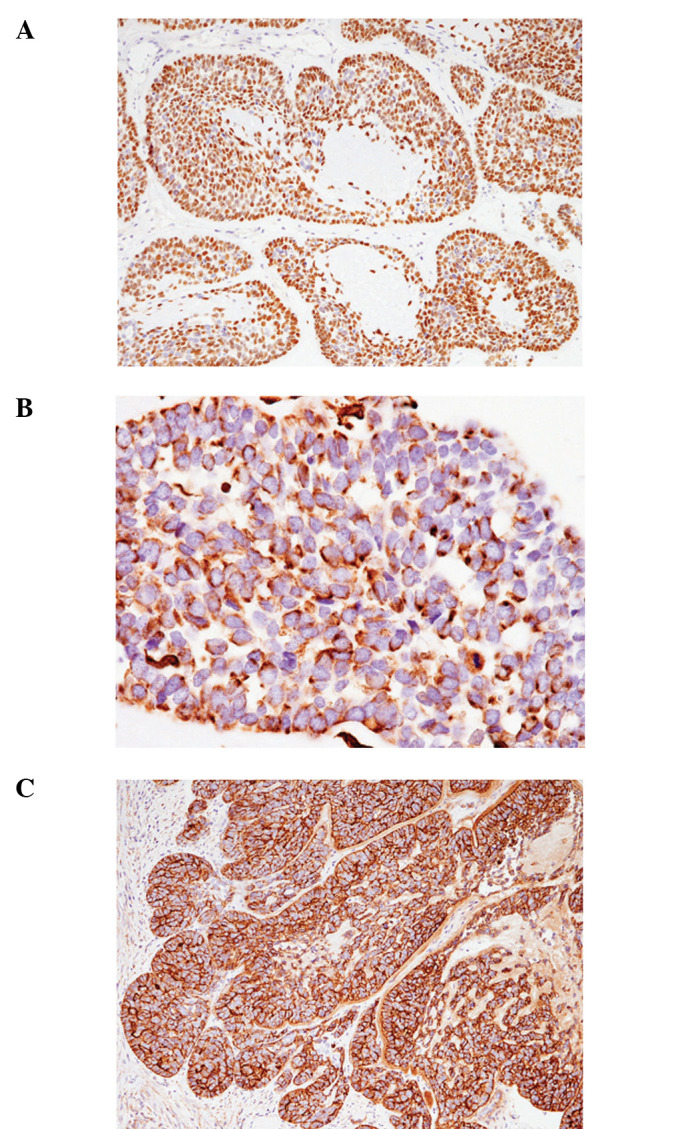
Immunohistochemical findings showing (A) p63 diffusely expressed in the nuclei of the tumor cells (magnification, ×100); (B) Perinuclear dot-like vimentin expression in the tumor cells (magnification, ×400) and (C) cathepsin K diffusely expressed in the tumor cells (magnification, ×100).

Table I shows the immunohistochemical findings of cases 1 and 2, each revealing similar results. The characteristic findings were diffuse positive immunoreactivity for p63 (Fig. 4A), negative immunoreactivity for α-smooth muscle actin and perinuclear dot-like positivity for vimentin (Fig. 4B). In addition, cathepsin K was diffusely expressed in each of the two cases (Fig. 4C).

## Discussion

In the present study, two cases of BSCC of the maxillary sinus are described. The clinicopathological features of the 26 previously reported cases of BSCC of the nasal cavity, as well as the 2 present cases, are shown in Table II. BSCC mainly affects the elderly (particularly individuals between 60 and 80 years of age), although it may occur, albeit rarely, in young adults. A comparison of all 28 cases showed that males are more commonly affected (male/female 18:10) and that the most common clinical symptoms are nasal obstruction, epistaxis and nasal tumors. The prognosis of BSCC of the nasal cavity is poor; 14 of the 28 reported cases succumbed to the disease and only seven were free of tumors following treatment. BSCC of the head and neck region shows aggressive clinical behavior [frequent lymph node metastases (62.5%), high mortality rate (47.5%) and poor three- and five-year overall survival rates (50% and 38.5%, respectively)] (10). Additionally, the present survey of the clinicopathological features of BSCC of the nasal cavity in all reported cases revealed that it has an aggressive clinical course, which corresponds to that of BSCC of the other head and neck regions.

The main histopathological differential diagnostic consideration for BSCC is adenoid cystic carcinoma (particularly the solid variant) since these tumors are also composed of basaloid cells and may have areas with a cribriform growth pattern (11). The main histopathological characteristics of BSCC that aid in distinguishing it from adenoid cystic carcinoma are greater nuclear pleomorphism, evidence of squamous differentiation, presence of necrosis and abundant mitotic figures (11). Moreover, immunohistochemical analyses are also useful for differentiating between these two diseases. The majority of adenoid cystic carcinomas show positive immunoreactivity for smooth muscle actin, but this marker is negative in BSCC (11). Moreover, p63 is diffusely expressed in the basaloid cells of BSCC, while this protein is only observed in the peripheral cells of adenoid cystic carcinoma (12). In addition, perinuclear dot-like vimentin expression is characteristic of BSCC, in contrast to the diffuse cytoplasmic expression of adenoid cystic carcinoma (11). In the present two cases, the histopathological features, including nuclear pleomorphism, evidence of squamous differentiation and frequent mitotic figures, and the immunohistochemical characteristics (diffuse p63 positivity, perinuclear dot-like vimentin expression and smooth muscle actin negativity) led to the final diagnosis of BSCC of the maxillary sinus.

To further identify the markers of this devastating disease, the expression of cathepsin K, a cysteine protease with marked collagenolytic and elastolytic activities, was investigated. Cathepsin K cleaves multiple sites within the triple helix of collagen types I and III, as well as at extracellular regions, whereas other proteases are more limited in their proteolytic activities. Cathepsin K was first demonstrated to play a significant role in osteoclast-mediated bone resorption (13), and it has also been recognized that this protein is involved in the extracellular matrix turnover in certain organs (13). Previous studies have demonstrated a role for cathepsin K in malignant tumors in certain organs, including the breast, skin and lungs (14–18). The majority of malignant melanoma cases (10/12) showed marked cathepsin K expression in the tumor cells (15), while all 50 cases of cutaneous basal cell carcinoma also showed expression of this protease (16). These results suggest that cathepsin K expression in tumor cells contributes to tumor invasion (15,16). By contrast, the majority of squamous cell carcinomas of the skin exhibited no positive immunoreactivity for cathepsin K in the tumor cells (only 2/38 cases were weakly positive), while the peritumoral stromal cells were markedly positive for cathepsin K (19). Moreover, cathepsin K expression was noted in only 31% of the cases of esophageal invasive squamous cell carcinoma (being particularly confined to the relatively sparse cancer cells located externally in the tumor foci) (20). Diffuse cathepsin K expression was observed in the two present BSCC cases. Therefore, although only two cases were examined in the present study, diffuse cathepsin K expression may be a characteristic immunohistochemical feature of BSCC. Moreover, cathepsin K expression in BSCC may contribute to tumor invasion and its highly aggressive clinical course since this protein has marked collagenolytic and elastolytic activities. Additional clinicopathological analyses are consequently required to clarify these issues and potentially aid in the future treatment of BSCC.

## Figures and Tables

**Table I:** Immunohistochemical findings.

Target	Case 1	Case 2
Cytokeratin (AE1/AE3)	+	+
Cytokeratin (34betaE12)	+	+
Epithelial membrane antigen	+	+
p63	+	+
α-smooth muscle actin	-	-
Vimentin	+ (dot)	+ (dot)
Cathepsin K	+	+

**Table II:** Clinicopathological features of basaloid squamous cell carcinoma of the nasal cavity.

Case No.	Age/Gender	Location	Clinical symptom	Metastases/invasion	Outcome	Ref.
1	78/M	Maxillary sinus	Cheek swelling, pain	None	NED, 25 months	3
2	60/M	Maxillary sinus	Cheek swelling, diplopia	Orbit, skull base, lung	STD, 6 months	3
3	50/F	Nose	Dyspnea	None	AWD, 1 year	4
4	59/M	Nose	Epistaxis	Not available	NED, 1 year	4
5	67/M	Nasal cavity	Epistaxis	None	NED, 4 months	5
6	53/F	Nasal septum	Epistaxis	None	STD, 8 years	6
7	81/F	Nasal cavity	Obstruction	None	Alive, 3 years	6
8	69/M	Nasal cavity	Blurred vision	None	STD, 1 years	6
9	32/M	Nasal cavity	Obstruction	Brain	STD, 7 years	6
10	72/M	Nasal cavity	Obstruction	Brain	STD, 1 years	6
11	33/F	Sinus	Obstruction, diplopia	Bone, lung	STD, 1 years	6
12	41/F	Nasal cavity	Obstruction	Lung	AWD, 5 years	6
13	75/F	Sinus	Obstruction	Dura	AWD, 2 years	6
14	64/M	Nasal cavity	Nasal mass	None	STD, 1 years	6
15	79/F	Sinus	Sinusitis, headache	Bone, lung	STD, 1 years	6
16	56/M	Nasal septum	Nasal mass	Cervical lymph node	NED, 2 years	6
17	46/M	Nasal septum	Obstruction, epistaxis	None	AWD, 8 months	6
18	86/F	Nasal septum	Nasal mass	None	STD, 6 months	6
19	79/M	Nasal cavity	Nasal mass	None	NED, 1 months	6
20	86/M	Nasal cavity	Epistaxis	None	STD, 2 years	7
21	36/M	Nasal cavity	Epistaxis	None	AWD, 1.5 years	7
22	59/M	Maxillary sinus	Not available	Not available	STD, 1 years	8
23	47/M	Maxillary sinus	Not available	Not available	STD, 1 years	8
24	69/M	Maxillary sinus	Not available	Not available	STD, 2.5 years	8
25	48/M	Maxillary sinus	Not available	Not available	Alive, 3.5 years	8
26	58/F	Nasal cavity	Epistaxis, obstruction	None	AWD, 17 months	9
Present case 1	85/F	Maxillary sinus	Nasal tumor, exophthalmus	Orbit, dura	AWD, 10 months	
Present case 2	60/M	Maxillary sinus	Obstruction	Dura, lung, liver	STD, 18 months	

AWD, Alive with disease; STD, succumbed to disease; F, female; M, male; NED, no evidence of disease.
